# Geometric confinement is required for recovery and maintenance of chondrocyte phenotype in alginate

**DOI:** 10.1063/1.5006752

**Published:** 2017-10-09

**Authors:** Megan E. Cooke, Mark J. Pearson, Richard J. A. Moakes, Christopher J. Weston, Edward T. Davis, Simon W. Jones, Liam M. Grover

**Affiliations:** 1 School of Chemical Engineering, University of Birmingham, Edgbaston B15 2TT, United Kingdom; 2 Institute of Inflammation and Ageing, MRC Musculoskeletal Ageing Centre, QE Hospital, University of Birmingham, Edgbaston B15 2TT, United Kingdom; 3 Institute for Biomedical Research, Medical School, University of Birmingham, Edgbaston B15 2TT, United Kingdom; 4 The Royal Orthopaedic Hospital NHS Foundation Trust, Bristol Road South, Northfield, Birmingham B31 2AP, United Kingdom

## Abstract

Human articular chondrocytes lose their native phenotype when expanded in traditional monolayer cultures. As a consequence, hydrogel encapsulation has been investigated as a means to maintain the natural phenotype. Alginate has been widely used for cartilage engineering as it has been shown to enable the recovery of a native collagen type II expressing chondrocyte phenotype. This study has evaluated whether the capacity of the materials to maintain/revert the phenotype is due to the composition of the material or the physical entrapment provided by the gel. To achieve this, an alginate “fluid gel” (a shear-thinning structured gel system) was produced of identical chemistry to a traditionally gelled alginate structure. Both were seeded with passaged primary human articular chondrocytes. Chondrocytes in quiescent alginate showed the recovery of the native phenotype and a spherical morphology. Chondrocytes in alginate fluid gel were unable to maintain the recovered phenotype despite having a spherical morphology and were shown to have a lower level of entrapment than those in quiescent alginate. These findings indicate that geometric entrapment is essential for the maintenance of a recovered chondrocyte phenotype in alginate.

## INTRODUCTION

Osteoarthritis (OA) affects 25% of the over-50 population, which is expected to rise with the increasingly obese world population.^1,2^ OA is characterized by the degeneration of articular cartilage, narrowing of the joint space, and pathological changes to subchondral bone; it is a painful and disabling condition.^3^ At present, there is no cure for OA, and many patients will eventually require joint replacement surgery. The native cartilage matrix is rich in collagen type II, proteoglycans, and chondrocytes, the cartilage-matrix producing cells.^4^ Critically, articular cartilage is avascular and hence has a limited capacity for self-regeneration. Indeed, it is widely believed that in end-stage OA, the cartilage loss is irreversible. Furthermore, as an avascular tissue, the delivery of pharmacological entities to the cells of the cartilage, the chondrocytes, is highly challenging.

An alternative to the pharmacological approaches being investigated to prevent OA progression is the surgical repair of focal defects in cartilage tissue in patients with early-stage OA, via autologous chondrocyte implantation (ACI).^5^ This repair strategy is dependent on the expansion of chondrocytes *in vitro* before re-implantation into the defect site.^6^ Critically, expansion in 2D monolayer culture causes chondrocytes to rapidly de-differentiate, with a marked loss of their expression of type II Collagen, aggrecan, and proteoglycan, and an associated increase in the expression of type I Collagen.^7–9^ This loss of chondrogenicity leads to the formation of an inferior hyaline cartilage, rich in collagen type I, which has been reported to be functionally similar to the fibro-cartilage that is formed following micro-fracture surgery.^10^ Consequently, this means that the ACI process has been of limited success. Therefore, there is a need to develop an improved method for the expansion of chondrocytes *in vitro*, which can maintain the chondrocyte phenotype and thus promote regeneration of functional articular cartilage.

Hydrogels have traditionally been used in regenerative medicine to act as extracellular matrix mimics due to their high water content and high degree of tunability.^11,12^ One such hydrogel is alginate, a linear, un-branched polysaccharide derived from brown algae that has been used in drug delivery, wound healing, and tissue regeneration.^13^ Containing β-d-mannuronic acid (M) and α-l-guluronic acid (G) residues arranged either in blocks (GGG/MMM) or alternating sequences (GMGMGM),^13^ alginate undergoes ionotropic gelation upon introduction of divalent cations to form crosslinks between G residues resulting in an “egg-box” structure.^14–16^

Previously, alginate has been shown to maintain the chondrocyte phenotype and to drive bone marrow derived mesenchymal stem cells towards a chondrogenic phenotype.^17–20^ Whether this is due to the physical entrapment of the chondrocytes in a 3D scaffold or due to the chemical influence of alginate is relatively unknown. The formation of a cartilage matrix by the encapsulation of allogenic chondrocytes is a promising approach to produce cartilage for surgical implantation.

**FIG. 1.**
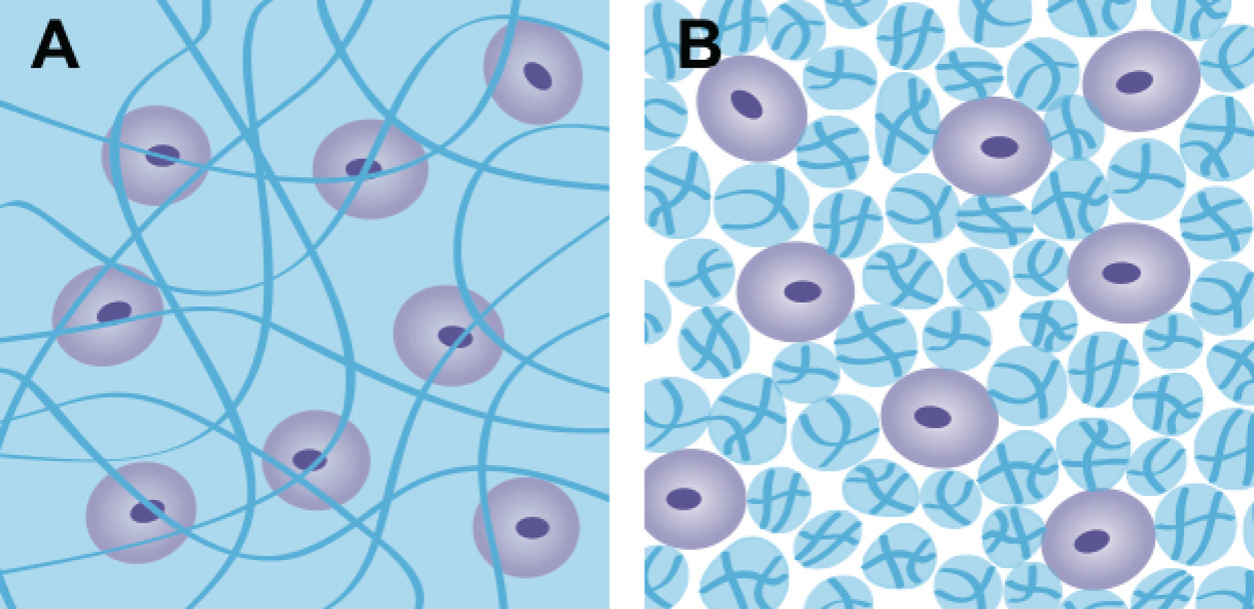
Schematic of cells (a) encapsulated in quiescent alginate and (b) suspended between fragmented alginate fluid gel.

In this study, the influence of the physical entrapment of alginate on the embedded chondrocytes has been investigated by comparing markers of cartilage matrix formation when chondrocytes are embedded in an alginate gel and between the particles that are formed in an alginate fluid gel. Fluid hydrogels are produced through the introduction of shear during gelation, resulting in the production of microparticles of gel.^21–24^ The microparticles interact with one another, and by doing so, they thicken in the absence of external shear stress and hence could be utilized in an injectable therapeutic solution for the delivery of chondrocytes to defects in articular cartilage. The cells dispersed within this structure are not physically entrapped as they would be in a normal, quiescently gelled structure; instead, they are suspended between fragments of crosslinked alginate (Fig. 1). They are, however, exposed to the same chemical stimuli. The aim of this study was therefore to determine the effect of different levels of encapsulation on the chondrocyte viability, morphology, and phenotype by comparing quiescent and fluid alginate.

## RESULTS

### Primary articular chondrocytes cultured in quiescently gelled alginate retain their viability and chondrogenic phenotype

**FIG. 2.**
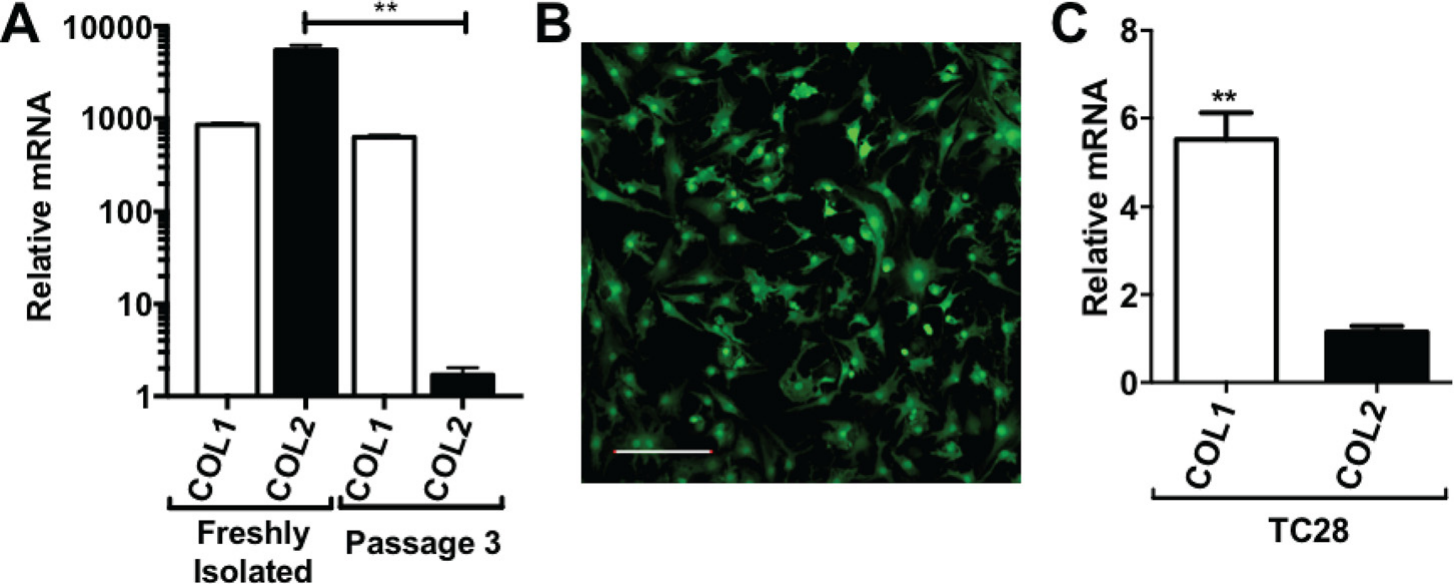
(a) Relative mRNA expression of collagen types I and II in primary human chondrocytes either directly after isolation from cartilage or following 3 passages in the monolayer. (b) Passage 3 human chondrocytes exhibiting a fibroblast-like morphology (scale bar (SB): 200 *μ*m). (c) Relative mRNA expression of collagen types I and II in the TC28 human chondrocyte cell line. **P < 0.005.

We first examined the effect of traditional monolayer culture on the chondrogenic phenotype of primary human articular chondrocytes (hACs). Freshly isolated (FI) primary hACs were found to express high amounts of type II collagen and low amounts of type I collagen, as determined by quantitative real-time polymerase chain reaction (qRT-PCR) analysis of COL2A1 and COL1A1 mRNA expression [Fig. 2(a)]. As expected, upon culturing in monolayer, primary hACs rapidly de-differentiated, adopting a more fibroblast-like morphology [Fig. 2(b)], and by passage 3, they exhibited significantly reduced (P < 0.01) expression of type II collagen [Fig. 2(a)]. Indeed, the expression profile of type I and type II Collagen in passaged primary hACs was similar to that observed in the chondrocyte TC28 cell line [Fig. 2(c)].

**FIG. 3.**
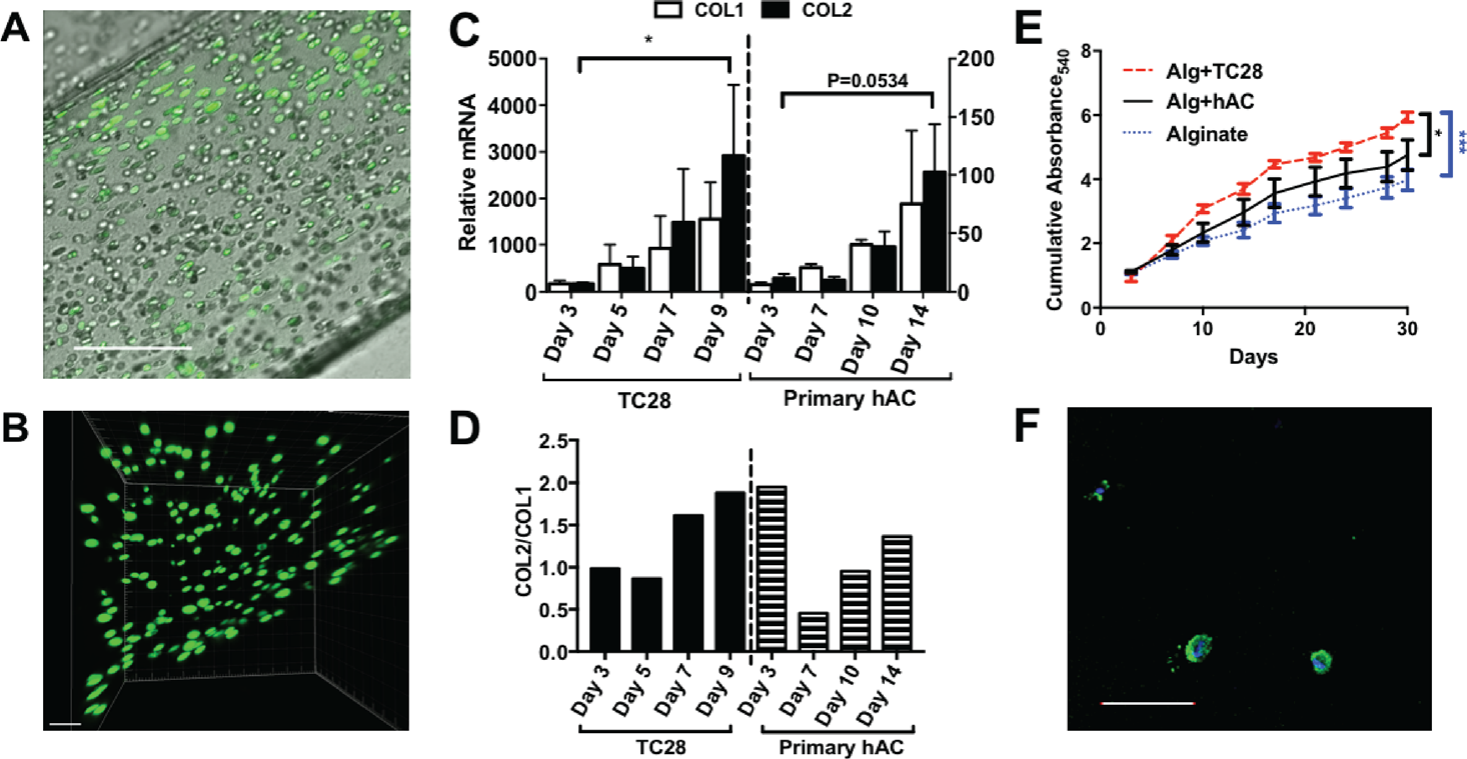
(a) Primary human chondrocytes encapsulated in quiescent alginate showing a flattened morphology at the edge of the construct and a spherical morphology in the centre of the construct, SB: 200 *μ*m. (b) z-stack demonstrating the cell morphology in 3-D, SB: 100 *μ*m. (c) Relative expression of collagen types I and II in TC28s and primary human chondrocytes (hACs) throughout 9 and 14 days of culture, respectively, with collagen type II to type I ratios (d). (e) Cumulative production of sGAGs as detected by the DMMB assay at 9 time points over 30 days; (f) evidence of aggrecan (green) in the cytoplasmic region with nuclei counterstained with DAPI (4′,6-Diamidino-2-Phenylindole) (blue), SB: 200 *μ*m. *: P < 0.05.

Next, we examined the effect of encapsulating primary hACs in quiescent alginate on both the cell viability and the chondrogenic phenotype over a period of 2 weeks. Following 14 days of encapsulation in alginate, cells in the scaffolds were stained using a live/dead stain (Calcein AM/ethidium homodimer-1 stain). Encapsulated primary hACs showed high levels of cell viability and displayed a flattened morphology at the edge of the constructs and a spherical morphology in the centre [Figs. 3(a) and 3(b)]. This is similar to chondrocytes in native cartilage, in contrast to the fibroblast-like morphology of primary hACs cultured in the monolayer. Analysis of COL2A1 and COL1A1 mRNA (messenger ribonucleic acid) expression over a time course of 14 days showed that primary hACs encapsulated in quiescent alginate did not de-differentiate since type II collagen expression was maintained throughout. Furthermore, encapsulation of the TC28 chondrocyte cell line in quiescent alginate suggests that they can be re-differentiated to a more native chondrocyte collagen phenotype [Figs. 3(c) and 3(d)]. In addition to measurements of collagen expression, the release of sulphated glycosaminoglycan (sGAG) side-chains was quantified over a time course of 30 days, as a marker of aggrecan proteoglycan turnover in the alginate scaffolds. Relative to alginate without cells, scaffolds containing encapsulated primary hACs or TC28s showed a significant accumulation of sGAG secretion over a period of 30 days, suggesting that alginate encapsulation supported and maintained aggrecan proteoglycan production [Fig. 3(e)].

The ability of quiescent alginate to support an anabolic chondrocyte phenotype, which produces cartilage extracellular matrix components, was then further validated by histochemical staining of alginate scaffolds. Aggrecan staining was observed in the cytoplasmic region of the chondrocytes [Fig. 3(f)].

### Formulation of a deliverable fluid alginate

**FIG. 4.**
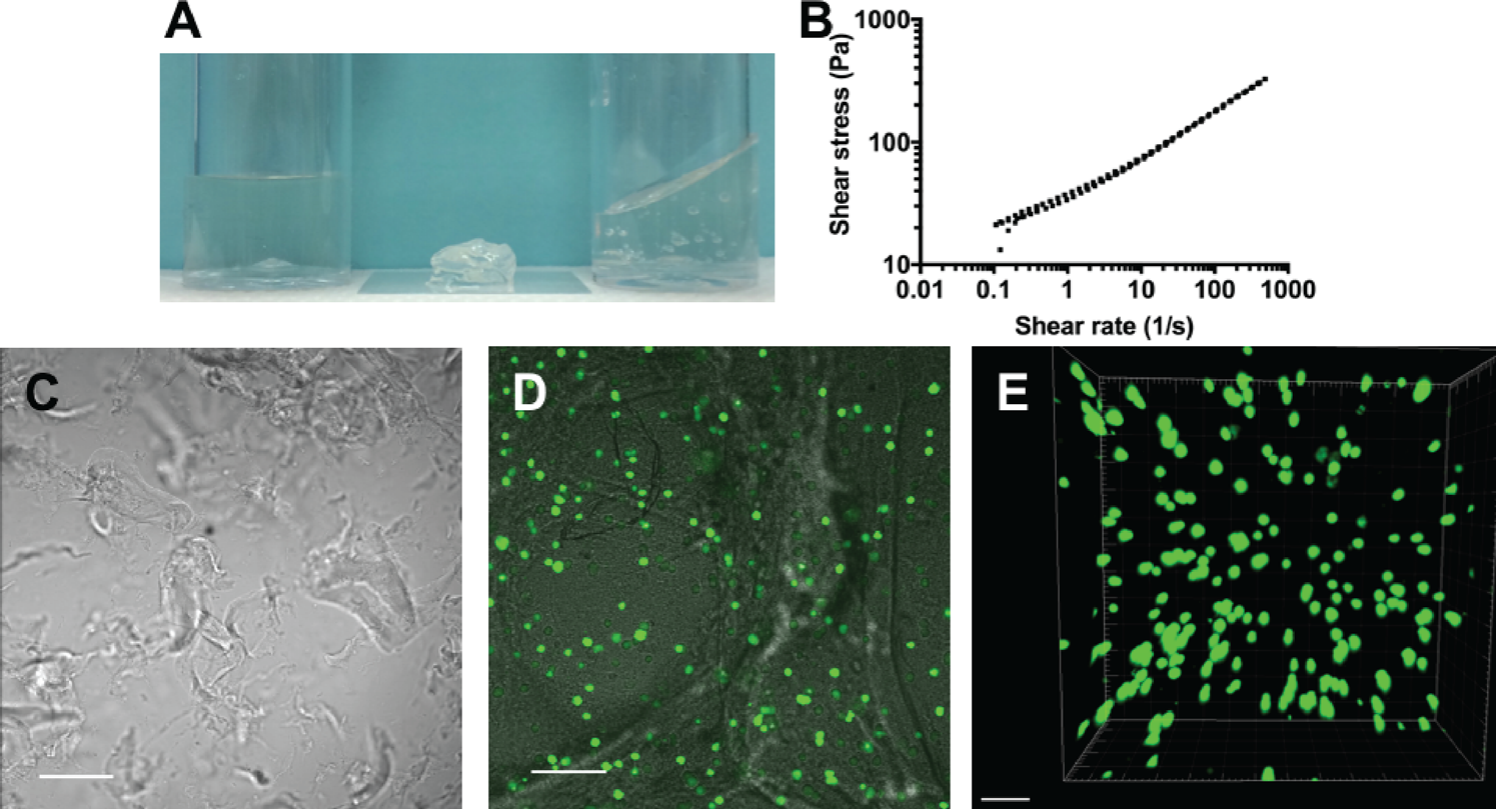
(a) Sol (left), quiescent (middle), and fluid (right) alginate; (b) change in shear stress with the shear rate over continuous ramps of the shear rate from 0.1-500-0.1-500 s^−1^; (c) fluid gel architecture (SB: 200 *μ*m); (d) fluid gel with chondrocytes showing the interaction with alginate fragments and ribbons (SB: 100 *μ*m); (e) z-stack of chondrocytes in fluid alginate 4 days after seeding, SB: 100 *μ*m.

An alginate fluid gel was produced by the drop-wise addition of CaCl_2_ to sol alginate under shear. The fluid alginate architecture is characterized by fragments connected by ribbon-like structures as shown in Fig. 4(c). Following shear ramps, the alginate fluid gel alginate showed a very small hysteresis loop, indicating that this fluid material will be stable during manipulation and handling [Fig. 4(b)]. After seeding of chondrocytes, they maintained a spherical morphology and showed high levels of viability [Figs. 4(d) and 4(e)].

**FIG. 5.**
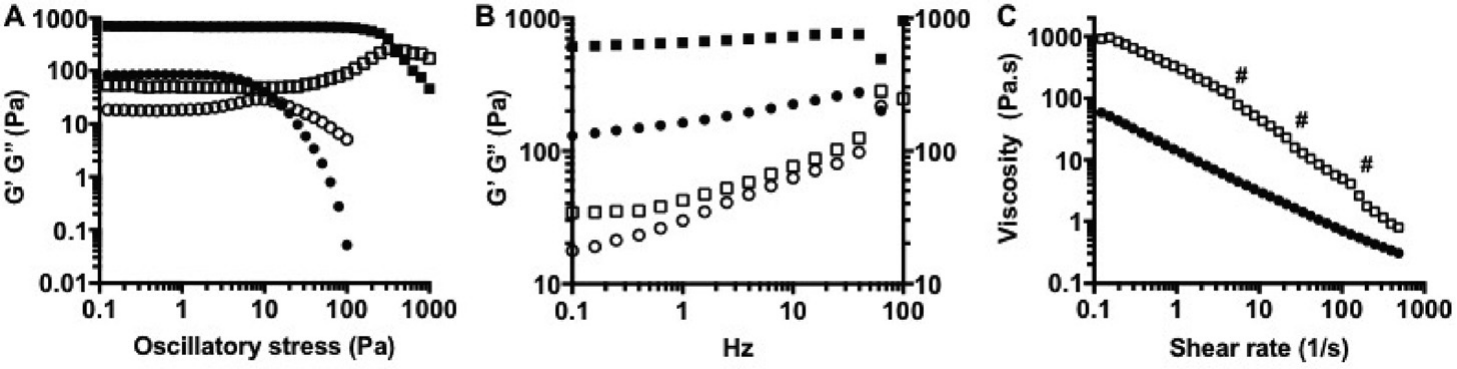
(a) Oscillatory stress sweeps (0.1–100 Pa, 1 Hz, 37 °C) showing the variation in the linear viscoelastic region (LVR). (b) Mechanical spectra (0.5% strain, 37 °C) showing the variation in the storage modulus (G′) and the loss modulus (G″). C: viscosity sweeps (0.001–100 s^−1^, 37 °C). (Quiescent alginate, squares; fluid alginate, circles; G′, filled symbols; G″, open symbols.)

**FIG. 6.**
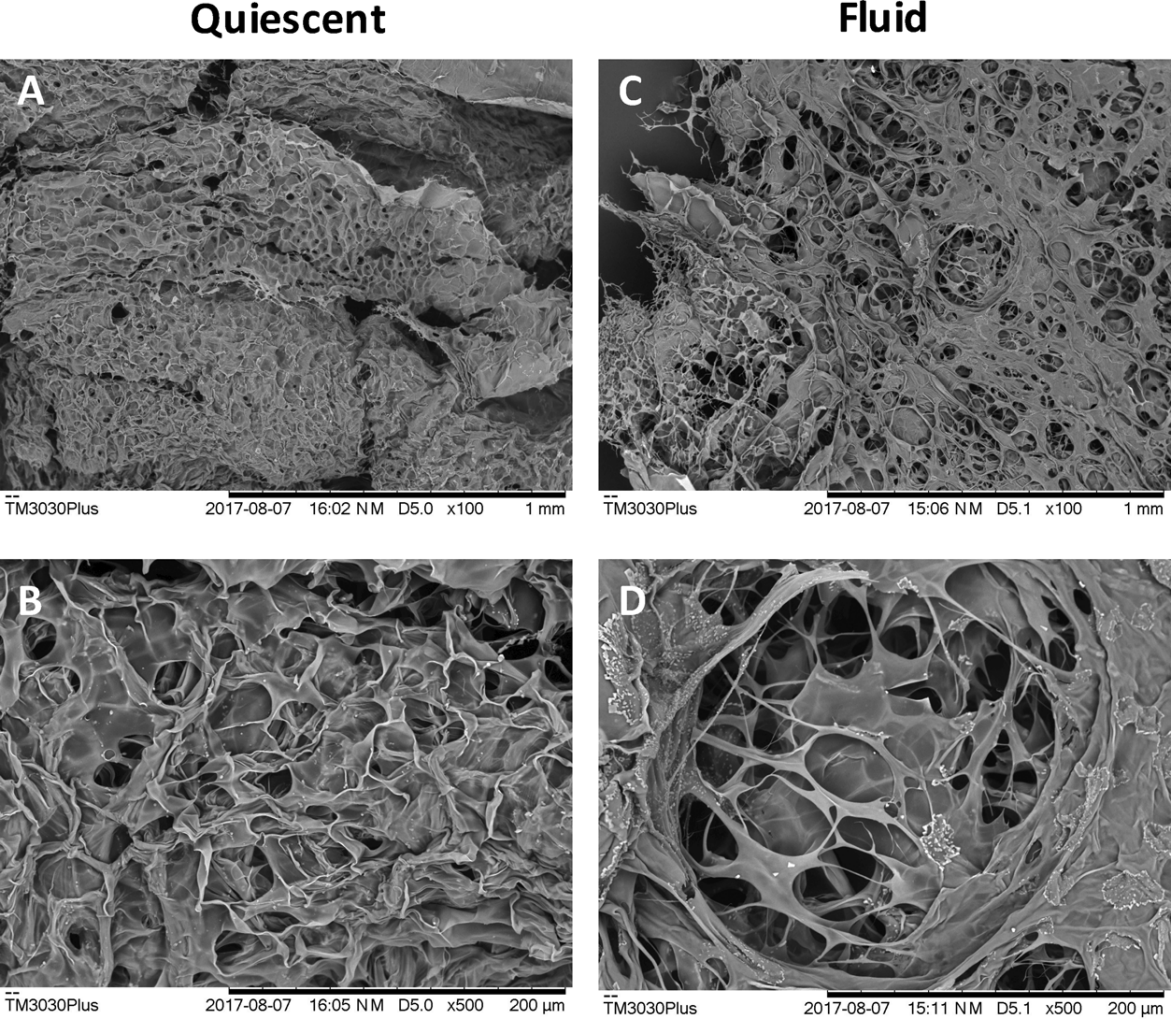
SEM images of thin sections of quiescent [(a) and (b)] and fluid [(c) and (d)] following freeze drying.

Dynamic small deformation rheology was performed to determine the viscoelastic properties of both the alginate quiescent and fluid gel systems. The introduction of shear during the gelation process resulted in a reduction in the storage modulus of fluid alginate, when compared to the quiescent gels prepared under static conditions [in the absence of shear (Table I)]. The presence of weak interactions and entanglements is confirmed through linear rheology [Fig. 5(b)], with the sheared alginate showing solid-like behaviours dependent on frequency, typical of weak gels. Interestingly, the quiescent gel shows a similar mechanical spectrum (at higher G′ values) inferring structures which mirror each other, complementing data obtained via microscopy images shown in Fig. 6. Changes throughout the gel microstructure were probed through stress sweeps [Fig. 5(a)], where shortening of the linear viscoelastic region (LVR) highlights the fluid gels' ability to flow upon being stressed. Fluid alginate (closed circles) shows strong shear thinning behaviour [Fig. 5(c)], where increasing the shear to the systems causes the reversible breakdown of the entangled polymers. However, this is not observed for the quiescent gel, as increasing shear to strains above the LVR causes fracturing of the gel resulting in artefacts that resemble thinning profiles [gel fracture points highlighted in Fig. 5(c) using “#”].

**TABLE I.** Storage (G′), loss (G″), and complex (G*) moduli of alginates.

	Quiescent			Fluid		
Hz/Pa	G′	G″	G*	G′	G″	G*
0.1	615.87	34.45	616.83	146.3	27.84	148.93
1	654.00	42.45	655.38	181.3	33.60	184.39
10	727.13	76.72	731.17	247.3	68.21	256.82

### Microstructural observations of fluid and quiescent alginate

To further understand the differences in the structure between the two materials, their microstructures were observed using scanning electron microscopy (SEM) following freeze drying. Fluid alginate was seen to have a less interconnected pore structure than quiescent alginate. Further, there were larger voidage regions observed throughout the fluid alginate structure, which were not seen in the quiescent microstructure (Fig. 6).

### Level of cell encapsulation in fluid and quiescent alginate

**FIG. 7.**
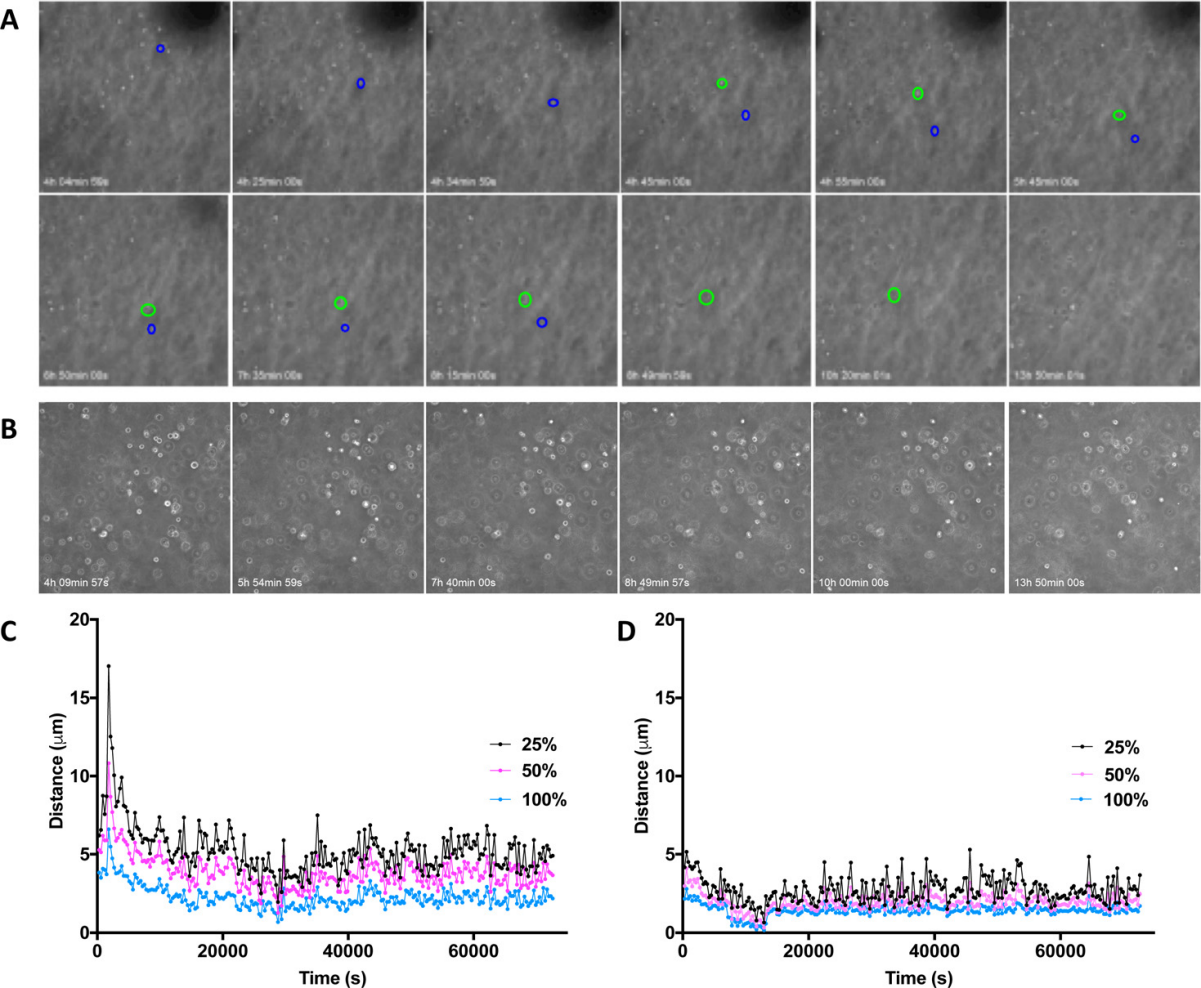
Tracking of chondrocytes in (a) fluid and (b) quiescent alginate over 14 h. Movement of cells is demonstrated by coloured circles. Quantification of chondrocyte movement in 2D in (c) fluid and (d) quiescent alginate. Multimedia view: https://doi.org/10.1063/1.5006752.110.1063/1.5006752.1; https://doi.org/10.1063/1.5006752.210.1063/1.5006752.2

To understand the level of encapsulation and confinement provided by alginate matrices of different structures, chondrocytes were imaged over time in culture. Individual chondrocytes in fluid gel were seen to move laterally across the field of view over a number of hours in the fluid alginate as well as downwards through the gel [Fig. 7(a), Multimedia view]. Chondrocytes in quiescent alginate, however, did not show as much movement over the same time-course [Fig. 7(b), Multimedia view]. Cell movement was quantified by frame-by-frame tracking, and then, the gel drift caused by contraction of the gel was normalized. Cells in fluid gel were found to move further per frame than cells in the quiescent alginate, particularly 25% most motile cells [Figs. 7(c) and 7(d)].

### The effect of alginate fluid gel on the phenotype of primary articular chondrocytes

**FIG. 8.**
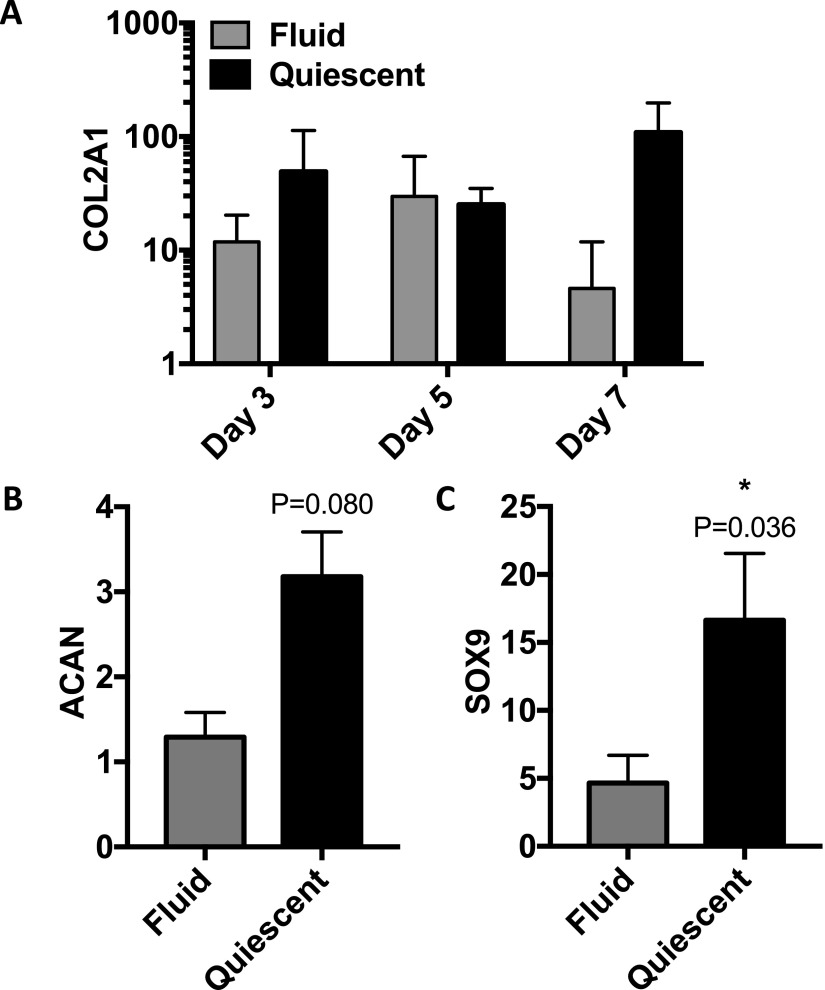
Relative mRNA expression of (a) collagen type II, (b) aggrecan (day 7), and (c) sox9 (day 7) in chondrocytes seeded in fluid and quiescent alginate.

To compare the efficacy of alginate fluid gel with that of quiescent alginate in supporting a chondrogenic phenotype, primary hACs were cultured in either alginate fluid gel or quiescent alginate for a period of 7 days. Following 7 days of culture, collagen type II and aggrecan expression were higher in the quiescent than fluid alginate, and sox9 expression was significantly higher in the quiescent alginate (Fig. 8).

### *Ex vivo* cartilage explant tissue promotes the chondrogenic phenotype of primary chondrocytes encapsulated in quiescent alginate

**FIG. 9.**
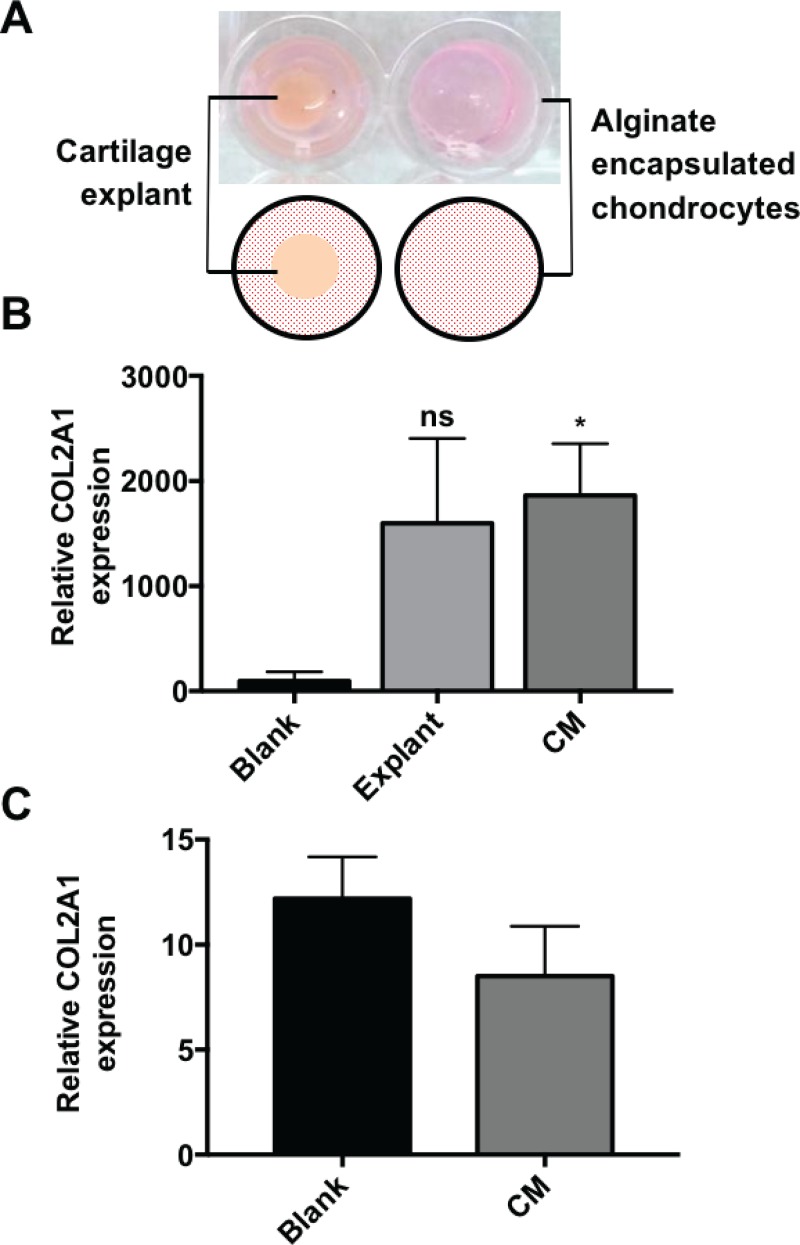
(a) Experimental set-up for explant (left) and blank/conditioned media (CM) (right). Relative collagen type II expression in (b) quiescent and (c) fluid alginate when cultured with basal media (blank), a cartilage explant, or conditioned media.

Due to the proximity of native cartilage tissue during the ACI repair of cartilage defects, *ex vivo* cartilage tissue and cartilage conditioned media (CM) were studied to determine their effect on the efficacy of using fluid and quiescent alginate as chondrocyte-carrying matrices. Following the encapsulation of a hAC population in quiescent alginate, the presence of a cartilage explant tissue [Fig. 9(a)] resulted in an increase in the expression of COL2A1 [Fig. 9(b)] following 14 days of culture. The effect of the physical presence of cartilage tissue was shown to be secondary to the factors released from it [Fig. 9(b)] since conditioned cartilage explant media resulted in a greater increase (P = 0.0376) in collagen type II expression than the cartilage tissue. This suggests that in the *in vivo* environment, alginate encapsulation of hACs will be even more efficacious in promoting and maintaining the chondrogenic phenotype due to the secretion of endogenous anabolic factors from the surrounding cartilage matrix. This condition was then investigated to determine if it could be used to drive the native alginate phenotype in fluid alginate culture. However, when comparing conditioned and basal media, there was no significant difference between the conditions [P = 0.3126, Fig. 9(c)]; this further supports that physical entrapment of chondrocytes is critical to maintenance of the native collagen type II phenotype.

## DISCUSSION

Alginate has been investigated for cartilage repair and regeneration strategies over the last three decades since it has been shown to support the redifferentiation of chondrocytes that have lost their native collagen type II and proteoglycan-producing phenotype.^27–29^ The mechanism of this effect is relatively unknown but has been attributed to the chondrocyte morphology, ligand adhesion, and matrix mechanical properties.^9,30^ Here, we aim to resolve the difference between the geometric and chemical influences of alginate.

The findings of this study support previous observations that chondrocytes are not phenotypically stable in 2D culture on untreated tissue culture plastic, with freshly isolated primary human knee OA chondrocytes rapidly losing collagen type II expression and adopting a fibroblast-like morphology within 3 passages. This is a well-known phenomenon in chondrocyte culture and has been attributed to the change in the morphology when cultured on a stiff substrate.^8,27,29^ In contrast, when primary chondrocytes were geometrically confined by encapsulation in quiescent alginate, they maintained native morphologies depending on the construct region, akin to native chondrocytes in articular cartilage tissue.^31^ Additionally, we found that primary human chondrocytes remained viable for up to 14 days of culturing in quiescent alginate constructs. Importantly, our data demonstrate that this geometric confinement of human chondrocytes in quiescent alginate promotes a chondrogenic phenotype with maintenance of collagen type II expression and aggrecan proteoglycan production. These data support the findings of Murphy and Sambanis who showed that the maintenance of the spherical chondrocyte morphology is necessary for the recovery of the chondrogenic phenotype following monolayer culture.^32^

Since the use of a quiescent alginate hydrogel construct for the regeneration of articular cartilage would require highly invasive surgery, we then examined whether alginate fluid gel, which would be more suitable for intra-articular injection, would exhibit the same chondrogenic promoting properties. Using the same alginate solution as that used to produce the quiescent constructs, we introduced shear during its gelation with the same concentration of crosslinking cations. This produced a self-supporting network of gel microparticles referred to as alginate fluid gel.^22,33^ This gelation process was highly reproducible, with little variation between batches (supplementary material, Fig. 1). Furthermore, rheological characterization demonstrated that with repeated frequency ramps, there was a consistent shear stress. These non-time-dependent shear-thinning properties suggest that such an alginate fluid-gel could be delivered by intra-articular injection without modification of its structural properties. Mechanically, alginate in its quiescent state was more elastic than fluid alginate and showed a higher complex modulus. The loss in the elastic structure of the fluid alginate is attributed to the presence of shear throughout the sol-gel transition, where mixing prevents a complete network from forming.^25,26^ There were also differences in the liquid-like and frequency dependent properties of the two materials. Both materials were stable at lower frequencies, with the fluid alginate showing a shorter LVR as it can flow when stressed. Such a phenomenon arises through the prevention of a completely gelled network, allowing polymer strands to move freely once the required energy has been met to yield any weak inter-particle interactions or entanglements.^21,25^

Following the investigation into the mechanical properties and the increased loss modulus of fluid alginate compared to quiescent alginate, the level of entrapment provided by the two alginate matrices was investigated. Using cell tracking, it was found that cells in fluid alginate were able to move laterally as well as vertically through the matrix, whereas in quiescent alginate, cells were confined to distinct regions. It was observed that there was increased movement of chondrocytes observed after the gel had “drifted.” Therefore, this effect may be due to fluid flow through larger pores and voids in the fluid alginate structure, which allow cells to move laterally through the gel network. This effect would also be likely observed in an *in vivo* situation due to joint motion. Further, the difference in the microstructure of the alginate may introduce local differences in the stiffness of the gel matrix, resulting in changes to cell behavior.

Despite the success of resuspending chondrocytes in alginate fluid gel, for cell viability to be maintained, our study has found that fluid alginate was not as effective as quiescent alginate in maintaining the chondrocyte phenotype upon culturing of constructs. This lack of effectiveness of fluid alginate to maintain and promote the chondrogenic phenotype of primary chondrocytes was even observed despite culturing hydrogels in the presence of explant conditioned growth media, which we had previously found significantly promoting the collagen type II expression of primary chondrocytes in quiescent alginate hydrogels. As such, physical entrapment provided by the quiescent alginate gel has been shown to be critical in the maintenance of a native chondrocyte phenotype.

## CONCLUSIONS

By producing a chemically identical fluid alginate, we have shown that with the lack of physical entrapment, passaged chondrocytes lose their ability to recover/maintain their phenotype. Even the introduction of growth factors released from cartilage which have a significant increase in collagen type II expression in quiescent alginate has no effect. We have demonstrated that maintenance of a spherical morphology alone is insufficient to recover a native collagen type II phenotype in passaged adult human chondrocytes. A certain level of geometric confinement for the chondrocytes is also essential.

## METHODS

### Tissue sources and isolation of primary cells

Joint tissue was collected from OA patients undergoing elective knee replacement surgery. Ethical approval was provided by the United Kingdom National Research Ethics Service (East of Scotland Research Ethics Service (11/ES/1044). All patients or their families provided consent. Full-thickness cartilage was excised from the femoral condyle and cartilage explant discs generated by cork-boring. For the isolation of primary human articular chondrocytes (hACs), cartilage was diced using a scalpel and digested in sterile-filtered 2 mg/ml collagenase type IA (0.5–3.0 FALGPA units/mg, Sigma Aldrich, Gillingham, UK) for 3 h. Both hACs and immortalised chondrocytes (TC28s) were cultured at 37 °C in a humidified incubator in growth media [DMEM (Dulbecco's Modified Eagle's medium) supplemented with 10% FCS (Fetal Calf Serum), penicillin-streptomycin (100 *μ*g/ml), 2 mM L-glutamine, and 1% non-essential amino acids, (Sigma Aldrich, Gillingham, UK)]. For the generation of conditioned media, a cartilage disc (diameter: 4.1 mm, depth: 2 mm) was incubated with culture media for 24 h before being applied to cultures.

### Alginate preparation

A solution of 1.5 wt. % medium viscosity alginate was prepared by the addition of dH_2_O to sodium alginate (Sigma) and heating to 85 °C for 1 h whilst stirring continuously. The solution was then allowed to cool to room temperature. Fluid alginate was prepared by applying a shear rate of 500 s^−1^; 10% 200 mM calcium chloride was added drop-wise, and the solution was equilibrated over 30 min at 20 °C (AR-G2, TA Instruments, New Castle, DE, USA). All alginate used for cell culture was autoclaved prior to use.

### Encapsulation of chondrocytes in alginate

For quiescent gels, both immortal chondrocytes (TC28) and primary hACs were mixed with 1.5 wt. % alginate to a concentration of 1 × 10^6^ cells/ml and seeded into the wells of a 96-well plate containing 0.2 M CaCl_2_ with or without the presence of cartilage explant tissue. The quiescent gel was then incubated for 20 min at 37 °C to allow crosslinking to occur. Gels were then rinsed twice with PBS (Phosphate-buffered saline), and 0.2 ml of chondrocyte growth medium was added and then incubated at 37 °C for up to 30 days. Conditioned media was generated from the 48 h incubation of femoral condyle cartilage explant tissue in chondrocyte growth media (15 ± 3 g in 30 ml of media). Alginate fluid gel was mixed with 1 × 10^6^ hACs and pipetted into a 96-well plate. Media were changed twice a week.

### Determination of chondrocyte viability

Cell viability in the alginate scaffolds was determined using a calcein AM/ethidium homodimer-1 LIVE/DEAD assay (Invitrogen). Calcein AM is cell-permeable and is hydrolysed to green fluorescent calcein by interaction with intra-cellular enzymes; cell-impermeable ethidium homodimer-1 binds to DNA and fluoresces red, and so, it binds to the DNA of dead/dying cells. Alginate constructs were imaged using a Leica DM 6000B confocal microscope.

### RNA isolation and quantification

Total RNA was isolated from alginate constructs and from chondrocyte monolayer cultures using TRIzol^®^ reagent (Life Technologies, UK). In brief, alginate constructs were homogenized in 1 ml of TRIzol reagent using a TissueRuptor (Qiagen). Chondrocyte monolayer cultures in 6-well plates were directly lysed with 1 ml of TRIzol reagent per well. Following the addition of TRIzol, 0.2 ml of chloroform was added and the samples were shaken vigorously for 15 s before being centrifuged for 15 min at 12 000*g* for the phase separation of RNA, DNA, and protein. The aqueous (RNA containing) phase was decanted, and RNA precipitated overnight at −20 °C by the addition of 0.5 ml of isopropanol. Following centrifugation, the RNA pellet was washed with ethanol, dried, and resuspended in 50 *μ*l of RNase-free H_2_O. RNA concentrations and A260/280 ratio were determined using spectrophotometry (NanoDrop 2000, ThermoScientific). RNA was stored at −80 °C prior to analysis by qRT-PCR.

### Quantitative real-time PCR

Precision OneStep SYBR Green Dye (PrimerDesign) was used with a β-actin reference gene (PrimerDesign) for quantification of the mRNA expression of COL1A1, COL2A1, ACAN, and SOX9 genes. The forward and reverse primer sequences were designed using Primer Express 3 software (Applied Biosystems) and produced by LifeTechnologies or PrimerDesign as indicated alongside primer sequences shown in supplementary material, Table I. Relative gene expression was determined by ddCT (delta-delta-cycle threshold), normalized to β-actin.

### Quantification of aggrecan proteoglycan

As a marker of aggrecan proteoglycan production, sulphated glycosaminoglycans (sGAG) released by the scaffolds into the supernatants were measured using a 1,9-dimethylmethylene blue (DMMB) assay.^34^ Supernatant samples (50 *μ*l) at 3, 7, 10, 14, 17, 21, 24, 27, and 30 days and standards of shark chondroitin sulphate dilutions were added to 200 *μ*l of DMMB colour reagent at pH 3. Alginate alone served as a control to remove the effect of interference of uronic acid groups in alginate. The absorbance was read immediately at A_540_ using a BioTek EL808 plate reader.

### Immunohistochemical analysis

For immunohistochemistry, cellular scaffolds were cultured for two weeks before analysis. Scaffolds were then fixed with 4% paraformaldehyde for 10 min before blocking in 0.1 M PBS with 10% goat serum and 0.3% Triton-X-100. Aggrecan was detected using the mouse anti-aggrecan primary antibody (1:200, ThermoFisher) and then Alexa-488 goat anti-mouse secondary antibody (1:500, Invitrogen). Nuclei were counterstained with DAPI (1:5000, Sigma Aldrich).

### Characterization of fluid and quiescent alginate

To visualize the “architecture” of the fluid gel, it was mixed at a 1:4 ratio with PEG (polyethylene glycol) to provide contrast between the phases and imaged using light microscopy (Leica DM 6000B).

Oscillatory rheology was used to determine the mechanical properties of fluid and quiescent alginate of the same chemistry. Discs (40 mm diameter) of quiescent alginate were produced using 0.2 M CaCl_2_ by the filter paper method described elsewhere.^35^ Fluid alginate samples were tested using a parallel, 40 mm sandblasted geometry, while quiescent alginate samples were prepared as 40 mm discs and tested using a ridged mesh as a gripper on flat 40 mm parallel plates on a AR-G2 Rheometer (TA Instruments, USA). A strain sweep was performed to determine the linear viscoelastic range of the gels. Storage (G′) and loss (G″) moduli were determined in a range of oscillatory frequencies (0.1–100 Hz) at 0.5% strain at 37 °C. The complex modulus (G*) was calculated by

$$G^{*}=\sqrt{{\left(G^{\prime }\right)}^{2}+{\left(G^{\prime \prime }\right)}^{2}.}$$ (1)

The angular frequency (ω) was calculated by

$$\omega =2\pi \left(\text{Hz}\right).$$ (2)

To determine any changes in the structure during experimental procedures, the response to shearing as experienced by fluids moving through a pipette tip was modeled by ramping the shear rate from 0.1–500-0.1–500-0.1 s^−1^ at 0.5% strain. Oscillatory stress ramps were performed to determine the LVR of each alginate matrix.

### Scanning electron microscopy (SEM)

1 mm thick sections of quiescent alginate and 1 mm smears of fluid alginate were snap frozen in liquid nitrogen and dried under vacuum for 48 h (Mini Lablyo, Frozen in Time, Ltd., York, UK). They were then mounted using conductive carbon tape and gold sputter coated. The topography was then imaged using a TM3030Plus benchtop SEM (Hitachi High Technologies, Schaumburg, USA) at 15 kV.

### Cell tracking

Cell tracking was used to determine the level of entrapment provided by the different alginate matrices. Chondrocytes were seeded in fluid and quiescent alginate at 1 × 10^6^ cells/ml and imaged every 5 min at 3 z-positions in each well over 20 h using a Cell-iQ and accompanying software Cell-iQ Imagen and Cell-iQ Analyser (Chip-Man Technologies, Tampere, Finland). Cell movement was quantified by tracking individual cells frame-by-frame using Cell-iQ Analyser. The trajectories were mapped, and the movement in 2D was normalized from gel drift (contraction) by tracking a reference point in each frame.

### Statistical analysis

Mean values and standard error of the mean are plotted. Unpaired t-tests were performed using Prism 7 (GraphPad software, CA, USA) with a significance of p < 0.05.

## SUPPLEMENTARY MATERIAL

See supplementary material for viscosity profiles of fluid alginate as it is produced as well as primer sequences and cell tracking videos.
